# 

**Published:** 1897-07-03

**Authors:** 


					?" UAUUUKY'S?\m~ CAD BUSY'S
COCOA m f?I! IT* COCOA
" The standard of highest purity at present
attainable,"? Lancet,
No. 562. Vol. XXIX. Cassia Sattjbday
July 3kd, 1897.
[SnbscriptionTemr,] pRICE TWOPENCE.

				

